# Earlier detection of melanoma using *in vivo* reflectance confocal microscopy—a retrospective analysis

**DOI:** 10.3389/fmed.2026.1826747

**Published:** 2026-06-16

**Authors:** Lea Pöschmann, Pauline Waibel, Frank Friedrich Gellrich, Stefan Beissert, Sarah Hobelsberger

**Affiliations:** 1 Department of Dermatology, Faculty of Medicine and University Hospital Carl Gustav Carus, TUD Dresden University of Technology, Dresden, Germany; 2 Skin Cancer Centre at the National Centre for Tumour Diseases and University Cancer Centre Dresden, Dresden, Germany

**Keywords:** confocal microscopy, detection, melanoma, nevus, non-invasive imaging

## Abstract

**Introduction:**

The incidence of melanoma has increased in recent decades. Non-invasive imaging devices, such as *in vivo* reflectance confocal laser microscopy (RCM), aid in the earlier detection of melanoma and decrease the number of patients that need treatment. RCM can visualise the skin to a depth of 200 μm at the cellular level.

**Methods:**

We conducted a retrospective analysis of melanocytic lesions, including invasive melanoma, *in situ* melanoma, and nevi, treated at the University Hospital Dresden. The control group (CG) included lesions examined solely through dermoscopy from 01 January 2016 to 31 December 2019, and the intervention group (IG) included lesions examined using both dermoscopy and, when necessary, RCM from 01 January 2020 to 31 December 2023. All melanoma diagnoses were confirmed histologically, while nevi were included based on clinical and histological diagnoses.

**Results:**

A total of 5,925 male and female patients (3,062 male and 2,863 female individuals0) with 6,651 melanocytic lesions were enrolled in the study, including 2,381 melanomas, 863 melanomas *in situ*, and 3,407 nevi. The lesions were found on the head and neck (1414), the trunk (1579), the upper extremities (925), and the lower extremities (892; for 54%, data were not collected). In the IG, significantly more invasive melanomas (43% vs. 30%, *p* < 0.001) and *in situ* melanomas (16% vs. 11%, *p* < 0.001) were diagnosed, while fewer nevi were diagnosed (42% vs. 60%, *p* < 0.001). Additionally, more melanomas were diagnosed at an early stage in the IG: IA (48% vs. 41%), *p* < 0.001. In the IG, fewer sentinel lymph node biopsies were performed (35% vs. 42%, *p* = 0.002), and there were significantly fewer positive results (21% vs. 27%, *p* < 0.001). Melanomas in the IG were less frequently ulcerated (15% vs. 21%, *p* < 0.001) and had a lower tumour thickness (1.81 vs. 1.97 mm, *p* < 0.001).

**Conclusion:**

Using confocal microscopy in routine care could lead to earlier detection of melanoma, including significantly more *in situ* and early invasive cases. Melanomas had fewer ulcerations, lower tumour thickness, and SLNBs were less frequently performed, as well as less frequently positive.

## 1 Introduction

The incidence of malignant melanoma in Germany has increased in recent decades, making it the fourth most common tumour (1). Early diagnostics, including skin examination and dermoscopy, are essential for a better prognosis (2). Dermoscopy provides an enlarged view of the skin lesions and offers greater specificity and sensitivity compared to visual examination. It allows for the visibility of special features such as vessels, dots, colour, and shape, which assist in identifying a lesion as malignant or benign (3, 4). The standard treatment for suspicious lesions is a surgical intervention with histological examination. This can initially be performed as a biopsy, shave excision, or complete excision with a delay of 1 or 2 weeks until the histological results are available. In particular, biopsies only sample a part of the lesion, which can affect the accuracy of the diagnosis (5).

Non-invasive imaging procedures can improve the early recognition of lesions and support clinical diagnosis. *In vivo* reflectance confocal microscopy (RCM) increases sensitivity and reduces the number of treatments needed for doubtful melanocytic lesions (6). By using an 830 nm diode laser, the epidermis can be visualised through reflection (7). RCM enables visualisation of the skin at a cellular level to a depth of 0.2 mm (8). Typical features of melanoma include pagetoid cells, nests of irregular melanocytes, and changes in the epidermis (6). The sensitivity rates for RCM are as high as 93%, with specificity rates reaching up to 76% (9, 10). Using RCM could reduce the number of treatments from 3.73 to 1.12 (11). RCM can be performed with either VivaScope 1,500 or VivaScope 3,000. The VivaScope 1,500 captures mosaic images that represent the entire lesion, while the VivaScope 3,000 can take stacked images (12).

RCM was introduced at the University Hospital Carl Gustav Carus Dresden in 2020, where it has been regularly used in doubtful cases since then. The present study aimed to whether the implementation of RCM in clinical practice resulted in the earlier detection of melanomas compared to the use of dermoscopy alone between 2016 and 2019, which served as a control group.

## 2 Materials and methods

### 2.1 Population sample and study design

A retrospective, monocentric, longitudinal study was conducted involving patients from the Department of Dermatology at the University Hospital Dresden, Germany. The study received approval from the ethics committee of the “Technische Universität” Dresden (reference number: BO-EK-475112023).

**Figure 1:**
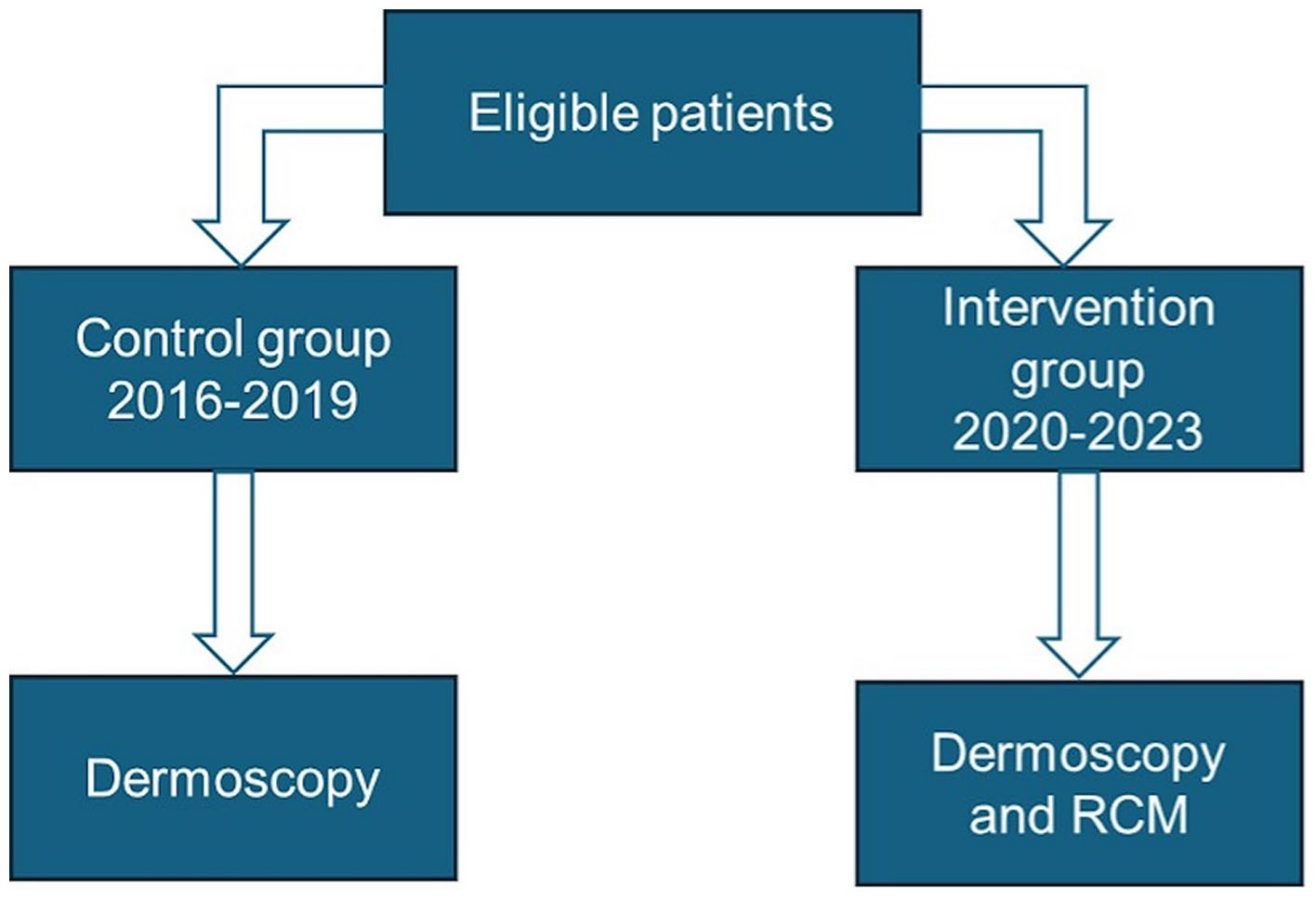
Patient enrolment for dermoscopy/dermoscopy and RCM.

Patients receiving routine care for melanocytic lesions were included in the study. These patients were divided into an intervention group (IG) and a control group (CG; shown in Figure 1). The CG included patients who had been diagnosed using dermoscopy only. Patients were enrolled in the CG if they had been examined between 01 January 2016 and 31 December 2019. The IG consisted of patients who were diagnosed using dermoscopy and, in doubtful cases, RCM. Patients were enrolled in the IG if they were examined between 01 January 2020 and 31 December 2023. RCM was conducted using a VivaScope® 3,000 (VivaScope GmbH, Munich, Germany). Dermoscopy was performed using a handheld dermatoscope.

In cases with a clear diagnosis, the decision to excise the lesion was made solely based on clinical dermoscopy. For doubtful lesions in the IG, the decision was made either through dermoscopy alone or by combining dermoscopy and RCM. All diagnoses of invasive and *in situ* melanoma were histologically confirmed. The histopathological examinations were performed in the Department of Pathology at the University Hospital Dresden. Nevi were included based on clinical and histological diagnoses, since excision was unnecessary for clinically benign nevi.

### 2.2 Data collection

The clinical-demographic data encompassed various patient-related characteristics, including the International Classification of Diseases (ICD) codes, localisation of the lesion, gender, age, and year of diagnosis. For melanoma patients, additional data were collected on tumour stage (AJCC stage and TNM stage), Clark level, sentinel lymph node (SLN) status, tumour thickness, ulceration, regression, and histological subtype. These data were collected using the hospital’s electronic documentation system (Orbis, Dedalus).

Eligible patients were classified using ICD codes (nevus D22.x, *in situ* melanoma D03.x, invasive melanoma C43.x, and melanoma of unknown origin C80.x).

Patients were included if they met the following criteria: ICD codes, gender, date of birth, age at diagnosis, and year of diagnosis.

For invasive melanoma, the following characteristics were analysed: tumour stage (AJCC stage and TNM stage), Clark level, SLN status, tumour thickness, ulceration, regression, and histological subtype. The exact location of the lesion was collected if available.

This study is presented in accordance with the STROBE checklist.

### 2.3 Inclusion criteria

Patients of all ages in routine care were included in the study. All melanocytic lesions examined between 01 January 2016 and 31 December 2023 were included. This included nevi, invasive melanoma (also mucosal melanoma), and *in situ* melanoma, all classified based on the ICD classification system.

### 2.4 Exclusion criteria

Uveal melanomas were excluded from the study.

### 2.5 Statistical analysis

All analyses were performed using R version 2023.06.1 + 524 with the packages psych, car, and rstatix and Microsoft Excel version 16.74. Demographic and clinical characteristics were analysed using descriptive statistics, which included mean, range, confidence intervals, and absolute and relative frequencies. We performed statistical tests to compare patient characteristics, clinical characteristics, and frequency of lesions between the two groups. Continuous variables were expressed as means and compared using the Mann–Whitney U test. Categorical variables were expressed as frequencies and compared using the Pearson χ^2^ test. A *p*-value of <0.05 was considered statistically significant.

## 3 Results

**Figure 2:**
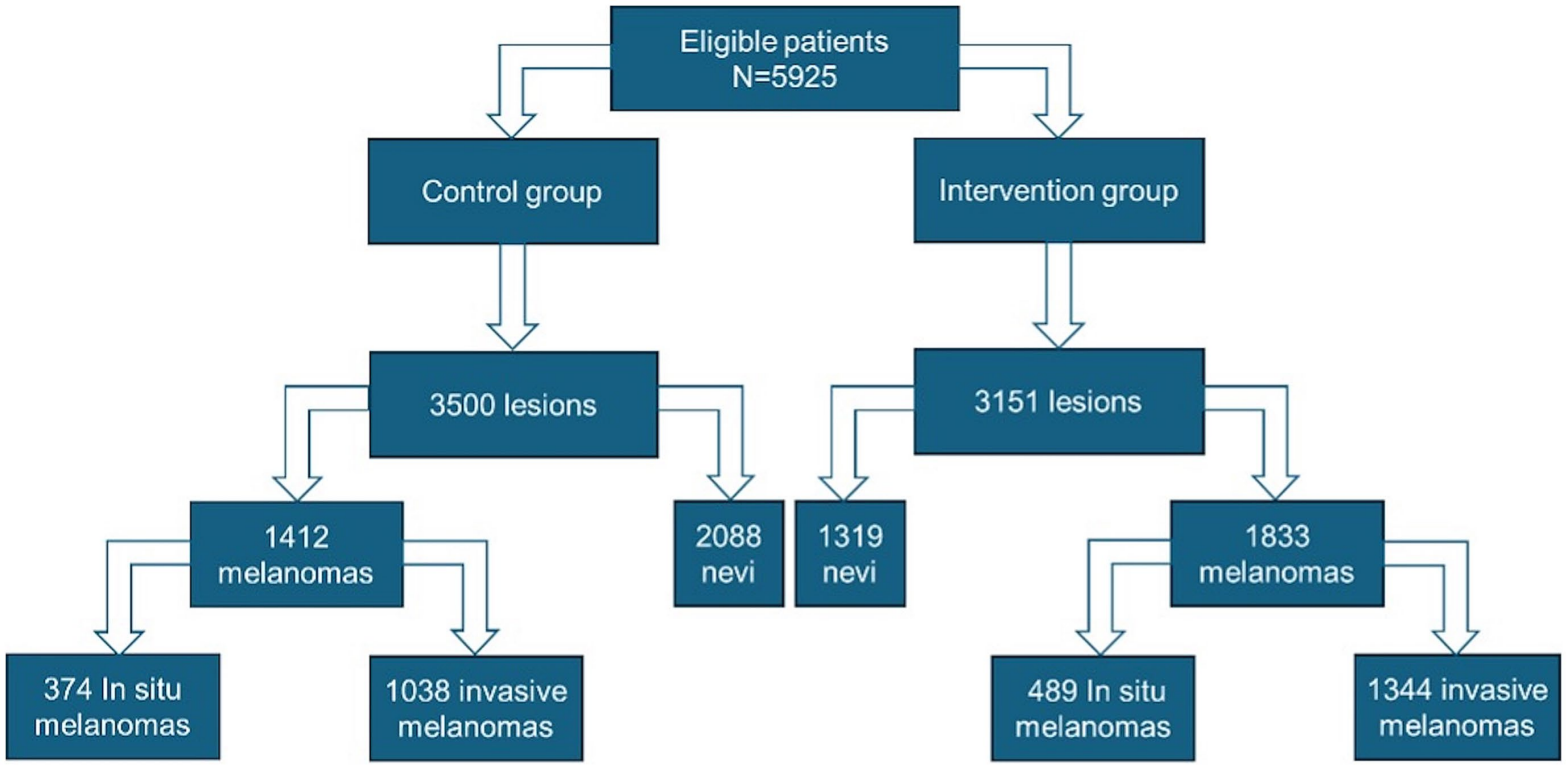
Number of included lesions.

The study included 5,925 patients (3,063 male and 2,862 female individuals) with a median age of 54.4 years, resulting in 6651 examined lesions. Patients in the CG had a median age of 51.5 years (range 0–98 years) and had 3,500 lesions examined, which included 1,038 melanomas, 374 *in situ* melanomas, and 2088 nevi (Figure 2). Patients in the IG had a median age of 57.7 years (range 0–96 years) and had 3,151 examined lesions, comprising 1,343 melanomas, 489 *in situ* melanomas, and 1,319 nevi. The majority of the lesions were localised to the trunk in both groups, with the head and neck being the second most common site (Table 1).

**Table 1:** Patient characteristics in the intervention and control group.

Characteristics	Control group	Intervention group	*p*-value
	*N* = 3,500 lesions	*N* = 3,151 lesions	
Sex			
Male	1823 (52.1%)	1,675 (53.2%)	0.3955
Female	1,677 (47.9%)	1,476 (46.8%)	
Median age (range)	51.5 years (0–98)	57.7 years (0–96)	<0.001
	95% CI 50.6–52.3	95% CI 56.8–58.5	
Localisation of lesion			
Head/neck	705 (20.1%)	709 (22.5%)	<0.001
Trunk	740 (21.1%)	857 (27.2%)	
Upper limb	423 (12.1%)	501 (15.9%)	
Lower limb	414 (11.8%)	478 (15.2%)	
Not applicable	1,218 (34.8%)	606 (19.2%)	

N, number; CI, confidence interval.

### 3.1 Patient characteristics

The control group comprised 52.1% male patients, while the intervention group had 53.2% male patients (Table 1). The majority of lesions were localised to the trunk in both groups, with the head and neck area being the second most common location (Table 1).

There were significantly more melanomas, as well as a higher percentage of melanomas diagnosed in the IG (*n* = 1,344) than in the CG (*n* = 1,038; 43% vs. 30%, *p* < 0.001; Table 2). Within the IG, significantly more melanomas were diagnosed as stage IA (48% vs. 41%, *p* < 0.001; Table 2). Advanced-stage melanoma was distributed similarly across both groups (Table 2). Tumour thickness differed significantly between the two groups (1.81 mm vs. 1.97 mm, *p* < 0.001; Table 2). Melanomas in the IG were less frequently ulcerated than those in the CG (14.3% vs. 18.9%, *p* < 0.001; Table 2). The most common type of melanoma in both groups was the superficial spreading melanoma (IG: 34.3% vs. CG: 34.9%), followed by nodular melanoma (IG: 8.8% vs. 19.8%) and Lentigo maligna melanomas (IG: 7.3% vs. CG: 11.4%; *p* < 0.001; Table 2). The localisation of the lesions was similar in both groups, with the majority of the lesions found on the trunk (*p* = 0.098, Table 2). Sentinel lymph node biopsies (SLNBs) were performed less frequently in the IG (35% vs. 42%, *p* = 0.002), and were less frequently positive (21% vs. 27%, *p* < 0.001; Table 2).

**Table 2:** Clinical characteristics of the melanocytic lesions.

	Control group	Intervention group	*p*-value
Invasive Melanomas n (%)	1,038 (29.7%)	1,343 (42.6%)	<0.001
Mucosal melanoma	2	3	
AJCC stage			
IA	421 (41%)	647 (48%)	<0.001
IB	212 (21%)	276 (21%)	
IIA	82 (8%)	105 (8%)	
IIB	86 (8%)	95 (7%)	
IIC	34 (3%)	35 (3%)	
IIIA	42 (4%)	22 (2%)	
IIIB	48 (5%)	23 (2%)	
IIIC	32 (3%)	91 (7%)	
IV	81 (8%)	49 (4%)	
Mean tumour thickness	1.97 mm (range 0.1–31 mm)	1.81 mm (range 0.08–51 mm)	<0.001
	95% CI 1.79–2.15	95% CI 1.64–1.98	
Ulceration	196 (18.9%)	192 (14.3%)	<0.001
Regression	64 (6.2%)	85 (6.3%)	0.9824
Histological subtype			
Superficial spreading	363 (34.9%)	462 (34.4%)	<0.001
Nodular	209 (19.8%)	162 (8.8%)	
Lentigo maligna	118 (11.4%)	98 (7.3%)	
Akrolentigineous	28 (2.7%)	36 (2.7%)	
Amelanotic	21 (2.0%)	12 (0.9%)	
Desmoplastic	7 (0.7%)	15 (1.1%)	
MUP	62 (6.0%)	32 (2.4%)	
Nevoid	26 (2.5%)	8 (0.6%)	
Spitzoid	10 (1%)	8 (0.6%)	
Epithelioid cell	1 (0.1%)	9 (0.7%)	
Polyploid	7 (0.7%)	13 (1%)	
Spindle cell	7 (0.7%)	13 (1%)	
Not applicable	179 (17.2%)	475 (35.4%)	
Localisation			
Head/neck	164 (15.6%)	230 (17.1%)	0.098
Trunk	343 (32.9%)	486 (36.2%)	
Upper limb	265 (25.5%)	338 (25.1%)	
Lower limb	211 (20.1%)	270 (20.1%)	
SLN performed	408 (39.3%)	459 (34.2%)	0.002
Positive	109 (26.7%)	96 (20.9%)	<0.001
Negative	299 (73.3%)	363 (79.1%)	
*In situ* melanomas	374 (10.7%)	489 (15.5%)	<0.001
Localisation			
Head/neck	190 (50.8%)	200 (40.9%)	
Trunk	68 (18.2%)	82 (16.7%)	
Upper limb	42 (11.2%)	72 (14.7%)	
Lower limb	38 (11.0%)	68 (13.9%)	
Nevi	2088 (59.7%)	1,319 (41.9%)	<0.001
Localisation			
Head/neck	350 (16.8%)	279 (21.2%)	
Trunk	329 (15.8%)	289 (21.9%)	
Upper limb	115 (5.5%)	91 (7.0%)	
Lower limb	165 (7.9%)	140 (10.6%)	

Mm, millimetres; SLN, Sentinel node; CI, confidence interval. Data on tumour thickness were not collected for 68 (6.6%) melanomas in the CG and 53 (3.9%) melanomas in the IG due to missing data on histological examination. Data on ulceration were not collected in 85 (8.2%) melanomas in the CG and 58 (4.3%) melanomas in the IG due to missing data on histological examination. Data on regression were not collected for 87 (8.4%) melanomas in the CG and 58 (4.3%) melanomas in the IG due to missing data on histological examination. Data on localisation were not collected for 10.9% in the CG and 13.9% in the IG. Data on localisation not collected for 54.1% in the CG and 46.3% in the IG.

In the IG, significantly more *in situ* melanomas were diagnosed (16% vs. 11%, *p* < 0.001; Table 2). *In situ* melanomas were predominantly located in the head and neck region in both groups (Table 2).

In the IG, significantly fewer nevi were diagnosed (41.9% vs. 59.7%, *p* < 0.001; Table 2). Nevi were mostly located in the head and neck region and the trunk (Table 2). Data on localisation were not collected in 54.1% of cases in the CG and 46.3% of cases in the IG.

Regarding secondary or tertiary melanomas, an increasing incidence has been reported at our centre since 2020, particularly in the lower stages (see Table 3). In the IG, significantly more secondary/tertiary primary melanomas were diagnosed compared to the CG (3.1% vs. 7.9%, *p* < 0.001).

**Table 3:** Incidence of secondary and tertiary melanomas per stage per year.

AJCC stage	IA	IB	IIA	IIB	IIC	IIIA	IIIB	IIIC	IIID	IV	MUP/ non-cutaneous melanoma	Sum
2016	2	1	2	0	0	0	1	0	0	0	0	6
2017	6	1	3	1	0	0	0	0	0	0	1	12
2018	7	1	3	0	0	0	0	1	0	0	0	12
2019	2	0	0	0	0	0	0	0	0	0	0	2
2020	8	4	0	0	1	0	0	0	0	1	0	14
2021	20	3	1	0	0	0	0	0	0	0	0	24
2022	17	5	0	2	0	0	0	0	0	0	0	24
2023	27	9	2	1	1	1	1	2	0	0	0	44

MUP, Melanoma of unknown primary.

## 4 Discussion

The stage of melanoma at the time of initial diagnosis is crucial for predicting outcomes (2). Previous studies reported that RCM shows higher sensitivity in diagnosing melanoma (10, 13). In the present study, melanomas were diagnosed significantly more often as *in situ* or stage IA melanoma in the IG. Previous studies reported a reduction in the number of treatments after the use of RCM (14). Pellacani et al. reported that the additional use of RCM in suspicious melanocytic lesions showed a higher positive predictive value and a 43.4% reduction in the number of lesions to be excised (5.3 vs. 3.0) (14). Meta-analyses suggest that RCM improves the detection rate of skin cancer by 7.7%, with similar sensitivities being comparable between dermoscopy and RCM (88.1% vs. 93.5%) (13). However, the specificity was significantly lower with dermoscopy alone, showing values of 52.9% compared to 80.3% with RCM (13). For melanoma detection, the pooled sensitivity was 88.4% vs. 93.5%, and the specificity was 49.1% vs. 78.8% (13). In our study, the overall detection rates of melanoma in our study were notably high. The patient cohort included referrals from medical practices (due to unclear lesions and planned surgeries), alongside those undergoing in tumour follow-up at our clinic, which primarily includes high-risk patients. The percentage of invasive melanomas was higher than that of *in situ* melanomas in both groups. This may be because a relevant percentage of patients had been previously treated at outpatient practices where RCM was not available.

Furthermore, tumour thickness was significantly lower in the IG, and tumours were less frequently ulcerated compared to the CG. The CG had a higher occurrence of nodular melanomas, which can lead to increased tumour thickness and more ulceration. Overall, the IG had a larger number of patients with histologically non-defined melanoma subtypes, resulting in a lower incidence of specific melanoma types than in the CG. According to German guidelines, there is no obligation to specify the histological subtype of melanoma, nor to report on factors such as regression, association with nevi, or desmoplasia (15).

Consequently, nevi were diagnosed less frequently in the IG. These results imply that some nevi diagnosed clinically without histopathological examination in the CG may have been misdiagnosed. The lower number of nevi diagnosed in the IG indicates that there are fewer cases to excise.

No significant difference in the incidence of advanced melanoma stages was observed in our study. At these stages, lesions primarily exhibit invasive dermatoscopic features, making them easier to recognise, thus reducing the significance of RCM.

In the present study, not all patients underwent RCM examination; only cases that were considered doubtful were examined using RCM. Therefore, the availability of RCM represents a significant improvement in diagnosis in a real-world setting.

RCM appears to be an effective method for diagnosing difficult lesions, and incorporating into clinical routines would be advantageous. First, it is valuable for planning operations and avoiding unnecessary biopsies (14). Second, RCM can support skin examinations to identify secondary melanomas in patients with a history of melanoma, who generally have a higher risk of developing further melanomas (16). Nevertheless, RCM has limited depth, preventing the visualisation of deeper tumour nests, and RCM captures only a part of the lesion, which may result in false negative diagnoses. Acral and mucosal melanomas are rare subtypes; therefore, data on RCM are limited. However, with RCM, other features such as dendritic cells or pagetoid cells can be identified independently of the histological subtype (17, 18).

Furthermore, the use of RCM depends on the user’s experience; therefore, to minimise this bias, the device was only operated by experienced individuals. To date, RCM is an expensive device that is not currently available in routine care. Moving forward, it maybe be possible to combine other imaging methods, such as total body photography, with RCM to optimise the early recognition of melanoma in high-risk patients.

The analysis revealed a higher incidence of secondary and tertiary melanomas in the IG than in the CG. Patients with a history of melanoma are at significant risk of developing a secondary melanoma. Risk factors include older age and male sex (19, 20). The risk of developing secondary melanomas is highest 1 year after the primary diagnosis, with stabilisation occurring over subsequent years (20). Wiener et al. report an incidence rate of 6.7% for secondary melanomas after 10 years (19). According to figures from our study centre, RCM may help identify secondary melanomas. The number of secondary/tertiary melanomas may have been underestimated in 2019 due to the lack of certification for the tumour centre in this year. Overall, the incidence of melanoma is increasing, as is the number of cases at our centre, which suggests that there may also be an increasing incidence of secondary melanomas (21).

## 5 Limitations

The present study had several limitations.

As the analysis was performed retrospectively, the groups were not matched. Differences in age, sex, and localisation may have influenced the results. RCM with VivaScope 3,000 examines only part of the lesion, and no mosaic images can be acquired. The selection of this part depends on the examiner, so there might be false-negative diagnoses. As the nevi included histological and clinical diagnoses, the number of lesions may be under- or overrepresented. Nevertheless, the majority of nevi are diagnosed clinically in everyday clinical practice. Furthermore, the analysis of clinical and histological diagnoses of nevi was performed in both groups. In several cases, histopathologic criteria like tumour thickness, ulceration, regression, or subtype were not applicable due to missing information in the histopathologic report or due to diagnosis (e.g., in melanoma of unknown primary (MUP)). In addition, in multiple clinical diagnoses, the exact localisation was not documented in the patient report. Due to the retrospective nature of the study, the patients were selected by filtering the internal documentation system of the clinic by ICD codes. In many cases, nevi were primarily classified under ICD D22.9, particularly when there are more than one to excise. Missing data may have influenced the results. However, this issue was limited to small number of lesions within this large cohort of 6,651 lesions. As our study was not a controlled trial, other effects may have also impacted the results. To minimise these effects, the skin examination and follow-up process remained unchanged. The number of lesions in both groups was quite similar.

The exact numbers of RCM examinations were not collected for the IG, which limits the benefits of RCM.

Due to a long inclusion period, there are relevant confounders, including the experience of the examiners, the involvement of various investigators, different workflows, and referral behaviour in outpatient practices.

Additionally, as the analysis was monocentric, the generalisability of the findings is limited due to selection bias towards high-risk patients. For other settings, such as dermatological practices, further validation is needed.

## 6 Conclusion

In a real-world setting, the use of RCM in the workflow alongside dermoscopy is associated with significantly earlier melanoma diagnoses, allowing for the identification of more in-situ and early invasive melanomas. With RCM, melanomas are less frequently ulcerated and have a lower tumour thickness. SLNB are performed less frequently, with fewer positive results. In the IG, significantly fewer nevi were diagnosed, which leads to a reduced number needed for excision.

## Data Availability

The raw data supporting the conclusions of this article will be made available by the authors, without undue reservation.
